# Impact of pasireotide on lipid and glucose metabolism in patients with acromegaly: a systematic review and meta-analysis

**DOI:** 10.1007/s40618-025-02642-0

**Published:** 2025-07-07

**Authors:** Flavia Costanza, Christian Basile, Sabrina Chiloiro, Eva Hessman, Dimitrios Chantzichristos, Alfredo Pontecorvi, Emanuele Bobbio, Maria Fleseriu, Daniela Esposito

**Affiliations:** 1 Department of Medicine, Division of Endocrinology, Diabetes and Clinical Nutrition, Sahlgrenska University Hospital, Gothenburg, 413 45 Sweden; 2 Department of Internal Medicine and Clinical Nutrition, Institute of Medicine, Sahlgrenska Academy, University of Gothenburg, Gothenburg, 413 45 Sweden; 3 Pituitary Unit, Department of Endocrinology, Diabetology and Internal Medicine, Fondazione Policlinico Universitario Agostino Gemelli, Istituto di Ricovero e Cura a Carattere Scientifico (IRCCS), Rome, 00168 Italy; 4 Department of Medical and Surgical Translational Sciences, Università Cattolica del Sacro Cuore, Fondazione Policlinico Universitario Agostino Gemelli, Istituto di Ricovero e Cura a Carattere Scientifico (IRCCS), Rome, 00168 Italy; 5 Department of Advanced Biomedical Sciences, Federico II University of Naples, Naples, 80138 Italy; 6 Division of Cardiology, Department of Medicine, Karolinska Institutet, Stockholm, 171 77 Sweden; 7 Biomedical Library, Gothenburg University Library, University of Gothenburg, Gothenburg, 413 45 Sweden; 8 Department of Cardiology, Sahlgrenska University Hospital, Gothenburg, 413 45 Sweden; 9 Department of Molecular and Clinical Medicine, Sahlgrenska Academy, University of Gothenburg, Gothenburg, 413 45 Sweden; 10 Pituitary Center, Department of Medicine and Neurological Surgery, Oregon Health and Science University, Portland, OR 97239 USA; 11 Department of Internal Medicine and Clinical Nutrition, Institute of Medicine, Sahlgrenska Academy, Department of Endocrinology at Sahlgrenska University Hospital, University of Gothenburg, Blå Stråket 8, Gothenburg, 413 45 Sweden

**Keywords:** Acromegaly, Pasireotide, Dyslipidemia, Diabetes mellitus, Hyperglycemia, Meta-analysis

## Abstract

**Background:**

Pasireotide long-acting release (PasiLAR), a somatostatin multireceptor ligand, is effective in achieving biochemical control but can increase the risk of hyperglycemia in acromegaly. However, the impact of PasiLAR on lipid and glucose metabolism in patients with acromegaly has not been systematically studied. This systematic review aimed at synthesizing evidence on PasiLAR effects (as monotherapy or combination therapy with pegvisomant) on lipid and glucose metabolism in patients with acromegaly.

**Methods:**

MEDLINE, Embase, Cochrane Library, and Web of Science were searched for studies published between 2000 and 2024. Prospective and retrospective studies reporting metabolic outcomes before and under PasiLAR treatment for a minimum follow-up of 6 months. Two reviewers screened eligible publications (3441), extracted outcomes, and assessed risk of bias.

**Results:**

Nineteen studies (896 patients) were included in the meta-analysis. PasiLAR was associated with increased fasting plasma glucose (FPG) (mean difference [MD] 23.4 mg/dL, 95% confidence interval [95%CI] 18.8–28.1]) and glycated hemoglobin (HbA_1c_) (MD 0.5%, 95%CI 0.4–0.7). A higher frequency of diabetes mellitus (DM) was observed after treatment (odds ratio 3.7, 95%CI 2.9–4.7). No significant changes in triglycerides, total cholesterol, or low-density lipoprotein cholesterol (LDL-C), and a modest but significant increase in high-density lipoprotein cholesterol (HDL-C) were recorded (MD 6.2 mg/dL, 95%CI 1.4–10.9]).

**Conclusions:**

In this large meta-analysis, PasiLAR was associated with increased HDL-C, FPG, HbA1c, and frequency of DM in patients with acromegaly. There was no effect on triglycerides, total cholesterol, and LDL-C.

**PROSPERO registration number:**

CRD42024544686.

**Supplementary Information:**

The online version contains supplementary material available at 10.1007/s40618-025-02642-0.

## Introduction

Acromegaly is a chronic disease characterized by the overproduction of growth hormone (GH), resulting in elevated levels of insulin-like growth factor 1 (IGF-I). The GH excess directly impairs insulin signaling and promotes lipolysis, leading to higher levels of free fatty acids, which in turn enhance hepatic gluconeogenesis and decrease glucose uptake in peripheral tissues [1–4]. Patients with acromegaly therefore have an increased risk of diabetes mellitus (DM) which, in turn, may increase the risk of cardiovascular morbidity and mortality [5–9]. The higher free fatty acid levels determined by the GH excess levels are used for the synthesis of triglycerides (TGs) in the liver, promoting the production of very low-density lipoproteins [10, 11], while IGF-I reduces the esterification of free cholesterol into high-density lipoprotein (HDL) particles [11]. Hypertriglyceridemia and low levels of HDL-cholesterol (HDL-C) levels are therefore common in acromegaly. These abnormalities in glucose and lipid metabolism observed in acromegaly are associated with chronic inflammation, which may contribute to poor prognosis among these patients [11, 12].

Treatment options for acromegaly include pituitary surgery, medical therapies such as dopamine agonists, somatostatin receptor ligands, and pegvisomant (PegV) [13–16] as well as radiotherapy. Previous meta-analyses have shown that pituitary surgery and PegV seem to improve glucose metabolism in acromegaly, whereas octreotide and lanreotide (first-generation somatostatin receptor ligands) do not have a significant effect on glucose homeostasis [17–19]. Studies on lipid metabolism from the early 2000 s suggest that octreotide and lanreotide can decrease TG, low-density lipoprotein cholesterol (LDL-C), and lipoprotein(a) [Lp(a)] levels, and increase HDL-C levels by normalizing GH and IGF-I levels [20–22]. On the other hand, data on the effects of PegV on lipid profile in acromegaly is somehow controversial, with some studies showing an increase in total cholesterol (TC) and a decrease in Lp(a) levels [23, 24].

Pasireotide long-acting release (PasiLAR) is a second-generation somatostatin multireceptor ligand capable of binding to four of the five somatostatin receptors (SSTRs), with highest affinity for SSTR-5 [25, 26]. It is mostly considered a second-line medical treatment for acromegaly [13, 27], with recent data from real-world studies showing that over 60% of patients achieve biochemical control [28]. The tolerability of PasiLAR seems similar to first-generation SSTR ligands with the exception of a higher incidence and severity of hyperglycemia. PasiLAR negatively impacts glucose metabolism by reducing both insulin and incretin secretion while exerting minimal effects on glucagon secretion [1, 29–31]. However, the effects of PasiLAR on lipid metabolism have been sparsely studied.

A comprehensive assessment of the metabolic effects of PasiLAR in patients with acromegaly is still lacking. To address this knowledge gap, we conducted a systematic review and meta-analysis assessing the impact of PasiLAR treatment on glucose and lipid metabolism in patients with acromegaly.

## Methods

The protocol for this systematic review followed the recommendation of the Preferred Reporting Items for Systematic Review and Meta-Analysis Protocols (PRISMA) statement [32] and was registered in PROSPERO in May 2024 (registration number: CRD42024544686). The final manuscript is reported according to PRISMA guidelines.

### Search strategy

A comprehensive search strategy focused on the key concepts of PasiLAR and acromegaly was developed for PubMed by the librarian author (E.H.) in collaboration with the other authors. After being tested against a list of known articles, the strategy was translated to fit the search logic of the other databases. The final search retrieved 5438 records and was conducted in April 2024 in MEDLINE (PubMed, Ovid), Embase (Elsevier), Cochrane library (Wiley), and Web of Science (Clarivate). No language restrictions were applied but the search was limited to results from January 2000 to May 2024, as PasiLAR is a novel treatment for acromegaly. The search was rerun in September 2024, adding 189 unique records to the previous search results. The search strategy was reviewed for accuracy using the Peer Review of Electronic Search Strategies criteria [33] and the PRISMA extension for searching [34]. The full search strategy for all databases is shown in Supplementary Fig. S1. Prospective or retrospective studies assessing glucose and lipid metabolism in patients with acromegaly on treatment with PasiLAR with at least 6 months’ follow-up were included in the systematic review. Review articles, animal studies, editorials, letters, and case reports were excluded from this systematic review. If the eligible studies included overlapping populations, only the study reporting the outcome of interest in a larger cohort was included in the meta-analysis.

### Study selection

**Fig. 1:**
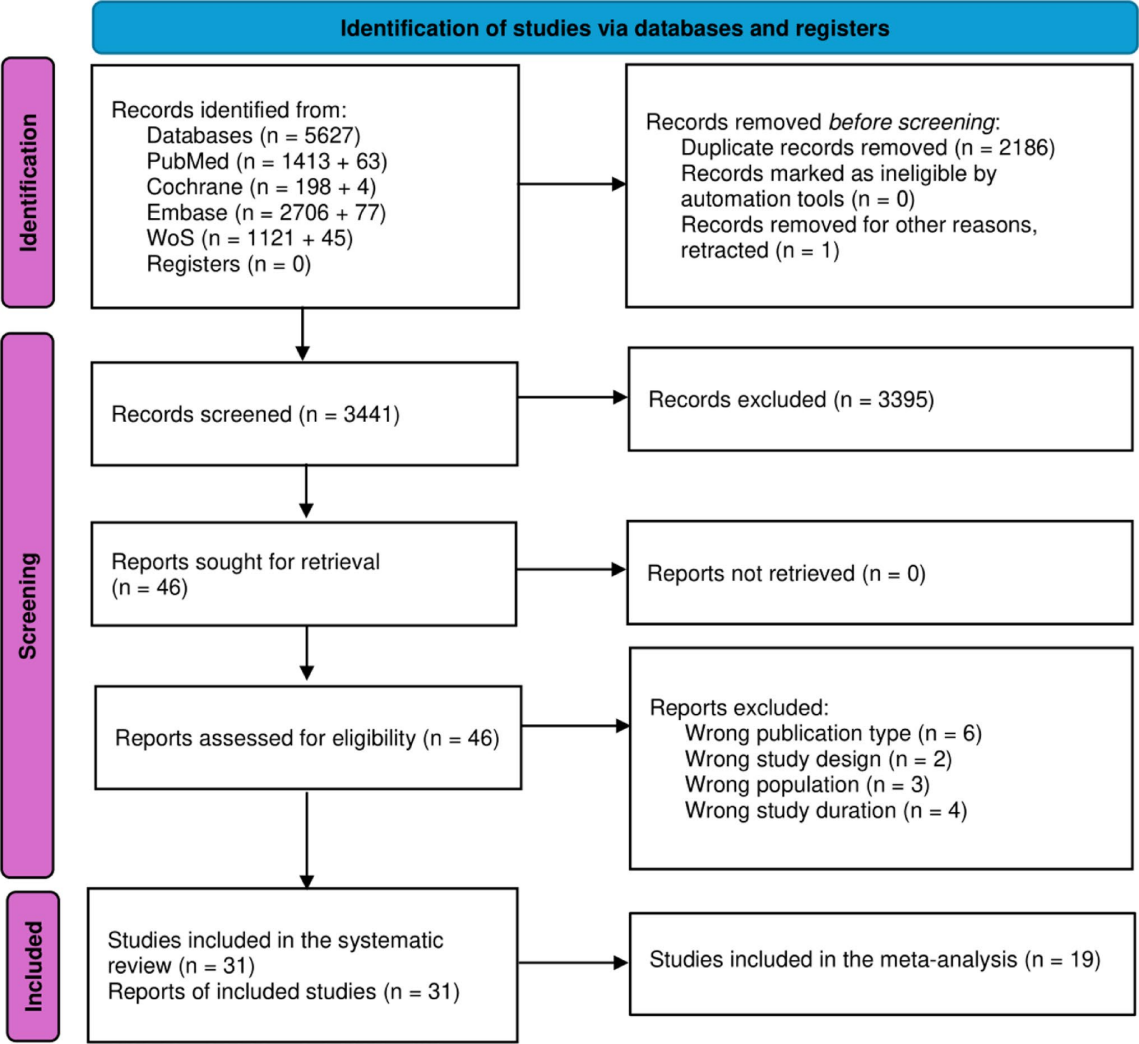
Study selection process according to PRISMA guidelines. Abbreviation: WoS, Web of Science

We selected all studies that met the following eligibility criteria: (i) randomized controlled trials, non-randomized prospective cohort studies, and retrospective cohort studies; (ii) PasiLAR alone or in combination with other acromegaly medications; (iii) at least 6 months of treatment with PasiLAR; and (iv) assessment of glucose and lipid metabolism parameters before and after PasiLAR or PasiLAR plus other acromegaly medications. Two reviewers (F.C. and C.B.) independently screened all identified titles and abstracts, and evaluated potentially eligible publications. Figure 1 shows the study selection process. Only articles considered relevant by both reviewers were selected for full-text evaluation. Agreement between the two reviewers was necessary for an article to be included at both the screening and full-text review phases. Disagreements in the study selection process were solved after discussion with a third reviewer (D.E.).

### Data extraction and quality assessment

Two reviewers (F.C. and C.B.) independently extracted the following data: study design, population size, data source, study period, mean treatment durations (months), acromegaly definition, age, ethnicity, geographic area, sex, PasiLAR as monotherapy or in combination with PegV, PasiLAR dose (mg/28 days), and percentage of patients with disease control expressed as normal IGF-I adjusted for age and sex. Table 1 shows the details of the included studies. Quality control checks on extracted data were performed by another investigator (D.E.), and all three reviewers involved in study selection and extraction independently assessed the risk of bias for all the studies based on the Cochrane risk-of-bias algorithm modified for non-randomized controlled trials by removing inapplicable criteria (Supplementary Table S1).

**Table 1:** Details of studies included for qualitative and quantitative analysis

First author, year (reference no.)	Study included in meta-analysis (yes/no)	No. of patients on PasiLAR (male/female)	Age, y (mean [SD] or median [IQR])	Mean PasiLAR dose (mg/28 days)	Disease control on PasiLAR (%)
*PasiLAR monotherapy*					
Colao et al. (2014) [60]	No	176 (85/91)	46 (18–80)	40	48.6
Gadelha et al. (2014) [40]	Yes	130 (57/73)	45.6 (19–82)	50	17.7
Petersenn et al. (2014) [56]	Yes	30 (14/16)	45.8 (25–65)	NA	50
Sheppard et al. (2015) [48]	No	74 (36/38)	46.5 (22–71)	NA	51.4
Chang et al. (2016) [61]	No	7 (NA)	NA	NA	85.7
Bronstein et al. (2016) [57]	Yes	81 (43/38)	45 (24–85)	NA	27.2
Schmid et al. (2016) [62]	No	130 (NA)	NA	50	NA
Fleseriu et al. (2017) [41]	Yes	44 (25/19)	45.5 (14.5)	40	NA
Tahara et al. (2017) [42]	Yes	33 (20/13)	52 (31–79)	38.7	27
Muhammad et al. (2018) [39]	No	15 (NA)	NA	60	93.3
Muhammad et al. (2018) [63] (extension)	No	31 (NA)	NA	49.6	93.3
Shimon et al. (2018) [43]	Yes	35 (20/15)	40.8 (13.3)	40	54
Lasolle et al. (2019) [45]	Yes	15 (5/10)	50 (27–67)	49.3	60
Colao et al. (2020) [64]	No	173 (NA)	NA	NA	NA
Gadelha et al. (2020) [44]	Yes	123 (61/62)	43 (22–76)	50	11.4
Masri-Iraqi et al. (2020) [46]	No	87 (43/44)	40.2 (11.3)	NA	NA
Akirov et al. (2021) [65]	No	19 (10/9)	48 (12.9)	41	79
Chiloiro et al. (2021) [38]	Yes	34 (13/21)	34.7 (11)	52.3	64.7
Samson et al. (2021) [47]	No	190 (NA)	41 (21–79)	40	NA
Witek et al. (2021) [49]	Yes	39 (16/23)	47.2 (12.7)	52	59
Stelmachowska-Banaś et al. (2022) [51]	Yes	26 (14/12)	42.6 (12.8)	55.3	38.5
Wolf et al. (2022) [50]	Yes	33 (13/20)	46 (13)	NA	58
Corica et al. (2023) [52]	Yes	21 (8/13)	56 (42–64)	36.6	81
Gadelha et al. (2023) [58]	Yes	50 (22/28)	40 (17–67)	44	54
Marques et al. (2023) [67]	No	50 (NA)	39 (17−67)	43	100
Ruiz et al. (2023) [37]	Yes	81 (42/39)	50 (1.5)	NA	58
Araujo-Castro et al. (2024) [59]	Yes	24 (NA)	44.7 (15.9)	40	70.8
Favero et al. (2024) [53]	Yes	19 (11/8)	48 (41–64.5]	47.3	73.7
Feldt-Rasmussen et al. (2024) [66]	No	190 (101/89)	42.5 (12.5)	40	NA
Pirchio et al. (2024) [54]	Yes	28 (10/18)	47.8 (12.1)	40.7	70.4
Urbani et al. (2024) [55]	Yes	50 (27/23)	43 (11)	44	67
*PasiLAR + PegV combined therapy*					
Chiloiro et al. (2021) [38]	Yes	6 (2/4)	39.5 (15)	60 + 21.6^a^ mg/wk	83.3

Abbreviations: IQR, interquartile range; NA, not applicable; pasilar, Pasireotide long-acting release; pegv, pegvisomant; SD, standard deviation; wk, week; y, year

^a^ PegV dose

### Study outcomes

The primary aim of this systematic review was to identify, critically appraise, and synthesize available data regarding the impact of PasiLAR treatment on glucose and lipid metabolism in patients with acromegaly. Specifically, the primary outcomes were differences before and after treatment with PasiLAR on fasting plasma glucose (FPG), glycated hemoglobin (HbA_1c_), and frequency of diabetes mellitus (DM). Secondary outcomes included the assessment of TGs, TC, HDL-C, and LDL-C.

### Data synthesis and statistical analysis

We extracted baseline and post-PasiLAR treatment mean and standard deviation (SD) or median and interquartile range (IQR) for continuous variables and proportion of binary outcomes. When the standard error was reported, the corresponding SD was calculated. If necessary, the outcomes of interest were converted to mg/dL (FPG, TG, TC, HDL-C, LDL-C) or percentage (HbA_1c_). In case of missing data for the SD, this was imputed using random forest. Thereafter, the mean difference (MD) between post- and pre-treatment values was computed for continuous outcomes, while odds ratios were calculated for binary outcomes. The meta-analysis was performed using both a fixed-effect and a random-effects model. Heterogeneity was evaluated using the chi-square test, quantifying inconsistency by *I*^*2*^ [35]. This describes the variability in effect estimates due to heterogeneity rather than chance, ranging from 0 (no heterogeneity) to 100 (maximum heterogeneity). This index was interpreted as follows: *I*^*2*^ 0% to10% indicated very low heterogeneity, *I*^*2*^ 10–50% indicated low-to-moderate, and *I*^*2*^ ≥ 50% indicated moderate-to-high heterogeneity. Publication bias was investigated by Egger’s test [36]. The estimated effect size and 95% confidence intervals (95%CIs) are reported. The significance level was set at 5%, two-sided. The meta-analysis was performed using R (version 4.3.3; R Core Team, 2024).

## Results

### Study selection

The literature search retrieved 3441 potentially relevant studies. Of these, 3395 were excluded based on title and abstract screening and 15 were excluded after full-text review (Fig. 1). Table 1 summarizes the total of 31 studies that were eligible for qualitative synthesis [37–67]. Considering overlapping population of patients with acromegaly to avoid double counting risk, only 19 studies were included in the quantitative analysis [37, 38, 40–45, 49–59]. Of these, only one study (38) included a group of patients on combination therapy (PasiLAR plus PegV).

### Study characteristics

Table 1 summarizes the characteristics of the 31 studies included in the systematic review. A total of 19 studies, encompassing 896 patients, were included in the meta-analysis. Study characteristics varied in: (i) daily dose (36.6–60 mg every 28 days); (ii) mean treatment duration (6–24 months); and (iii) previous treatments (neurosurgery and/or medical therapy and/or radiotherapy). Ten studies included in the meta-analysis were prospective randomized controlled trials (53%), while nine studies were retrospective (47%). Half of the studies were conducted in Europe (50%). Most patients were male (58%). Median age (IQR) was 45.8 (43.0–47.8) years.

### Glycometabolic outcomes

**Fig. 2:**
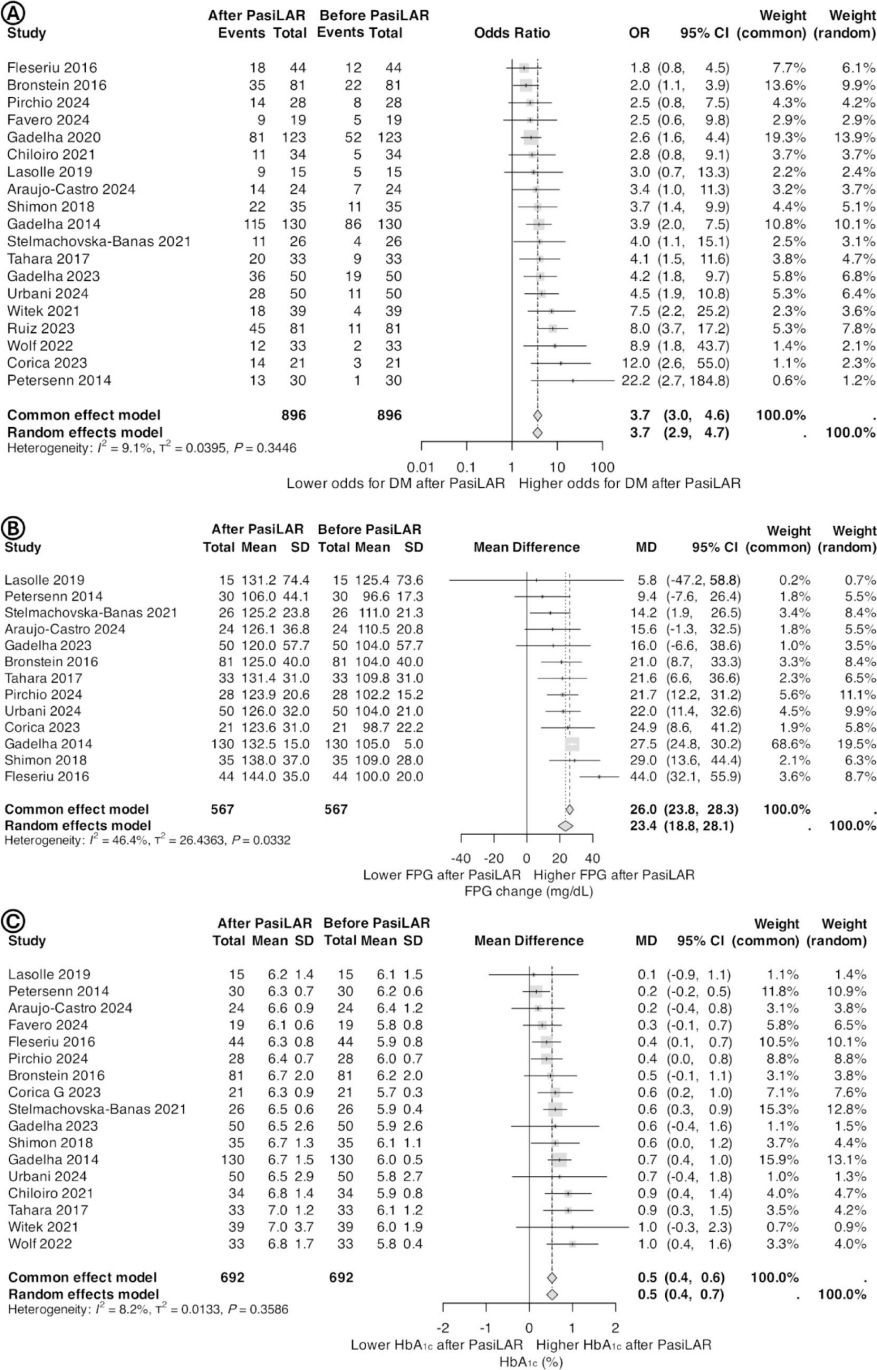
Pooled estimates for (**A**) odds ratio for diabetes mellitus, (**B**) mean difference in fasting plasma glucose, and (**C**) mean difference in glycated hemoglobin in patients with acromegaly treated with PasiLAR for at least 6 months (post-treatment versus baseline). Abbreviations: CI, confidence interval; DM, diabetes mellitus; FPG, fasting plasma glucose; HbA_1c_, glycated hemoglobin; MD, mean difference; OR, odds ratio; PasiLAR, pasireotide long-acting release; SD, standard deviation

For all of the included studies, the proportion of patients with DM at baseline was 30.9% compared to 58.6% after treatment with PasiLAR. Treatment with PasiLAR was associated with an increased risk of DM compared to baseline (odds ratio 3.7, 95%CI 2.9–4.7) with very low heterogeneity (*I*^*2*^ 9.1%) (Fig. 2A).

Thirteen studies, involving 567 patients, provided data on FPG. PasiLAR treatment was significantly associated with higher FPG levels (MD 23.4 mg/dL, 95%CI 18.8–28.1) with moderate-to-low heterogeneity (*I*^*2*^ 46.4%) (Fig. 2B).

Seventeen studies, comprising 692 patients, reported data on HbA_1c_ levels. PasiLAR treatment was associated with higher HbA_1c_ (MD 0.5%, 95% CI 0.4–0.7) with a very low heterogeneity (*I*^*2*^ 8.2%) (Fig. 2C).

Only one study [38] investigated the effect of PasiLAR + PegV combined treatment. In this study, four patients (66.4%) had DM at baseline, one patient (16.7%) impaired FPG levels, and one patient (16.7%) normal glucose metabolism. With combination treatment, glucose metabolism improved in three patients (50%), remained unchanged in two (33.3%), and worsened in one (16.7%). FPG data was only available at baseline. HbA_1c_ showed a reduction post-treatment (− 0.3%, SD 1) compared to baseline values (7.6%, SD 1.5). Due to the limited data available on combined therapy, a meta-analysis could not be performed.

### Lipid outcomes

**Fig. 3:**
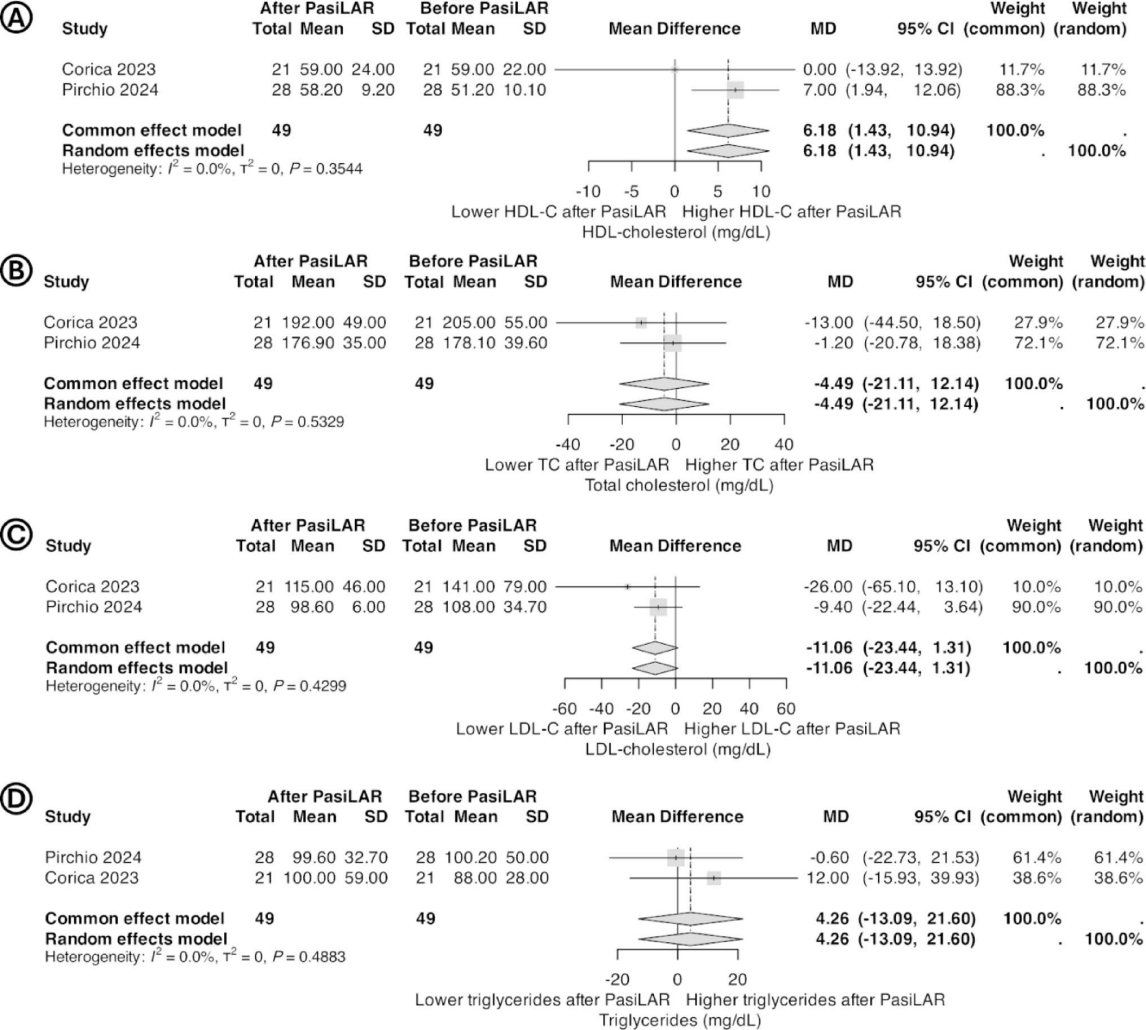
Pooled estimates for mean difference in (**A**) HDL-cholesterol, (**B**) total cholesterol, (**C**) LDL-cholesterol, and (**D**) triglycerides in patients with acromegaly treated with PasiLAR for at least 6 months (post-treatment versus baseline). Abbreviations: CI, confidence interval; HDL-cholesterol, high-density lipoprotein cholesterol; LDL-cholesterol, low-density lipoprotein cholesterol; MD, mean difference; PasiLAR, pasireotide long-acting release; SD, standard deviation

Two studies [52, 54] with 49 patients reported lipid parameters before and after PasiLAR treatment. PasiLAR was associated with a significant increase in HDL-C levels (MD 6.2 mg/dL, 95%CI 1.4–10.9; *p* < 0.001, *I*^*2*^ 0%) (Fig. 3A). No significant changes were observed for TC (MD − 4.5 mg/dL, 95%CI − 21.1–12.4; *I*^*2*^ 0%), LDL-C (MD − 11.1 mg/dL, 95%CI − 23.4 to 1.3; *I*^*2*^ 0%), or TGD (4.3 mg/dL, 95%CI − 13.1 to 21.6; *I*^*2*^ 0%) (Fig. 3B-D).

### Risk of bias and sensitivity analyses

**Fig. 4:**
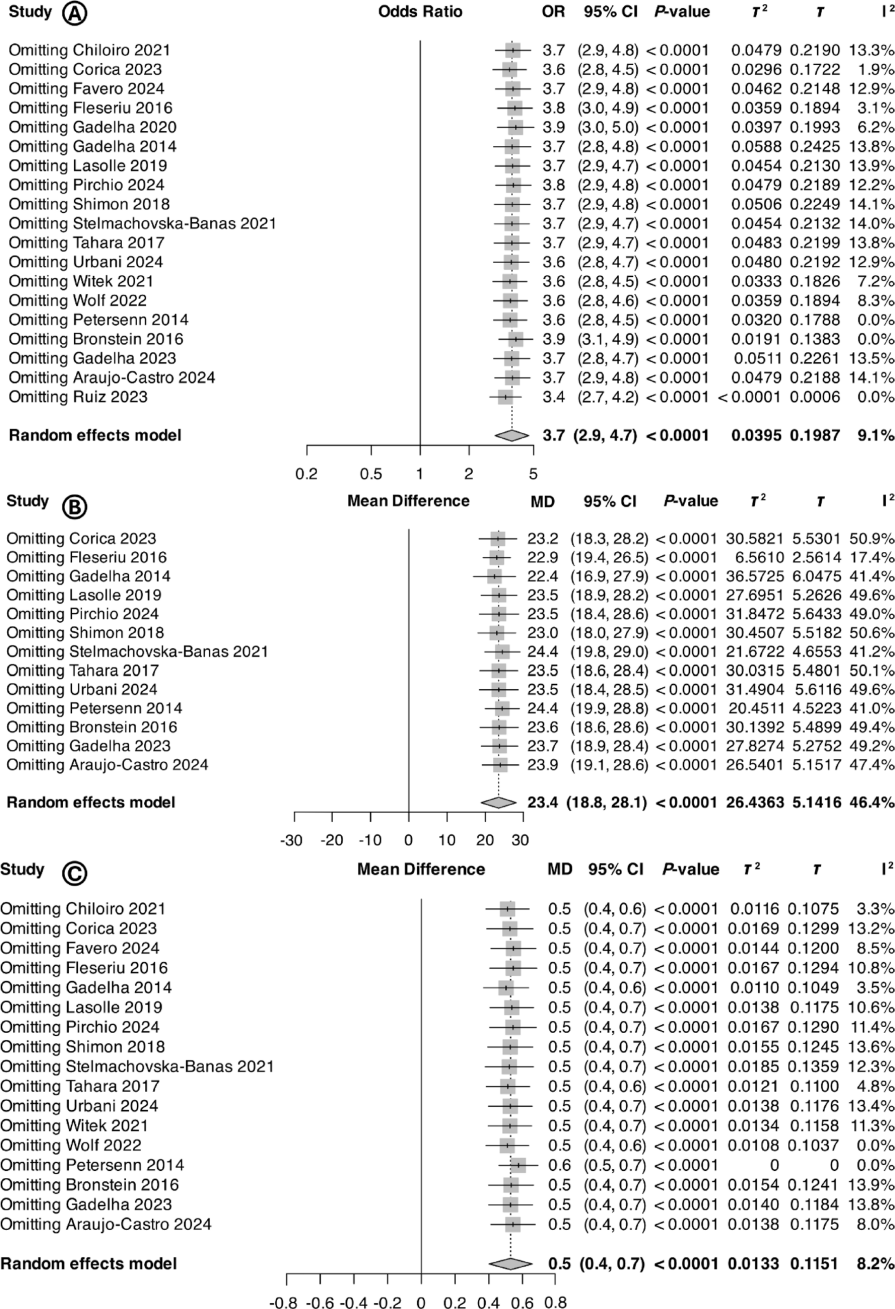
Leave-one-out meta-analysis showing consistent results for (**A**) odds ratio for diabetes mellitus, (**B**) mean difference in fasting plasma glucose, and (**C**) mean difference in glycated hemoglobin. Abbreviations: CI, confidence interval; OR, odds ratio; MD, mean difference

**Fig. 5:**
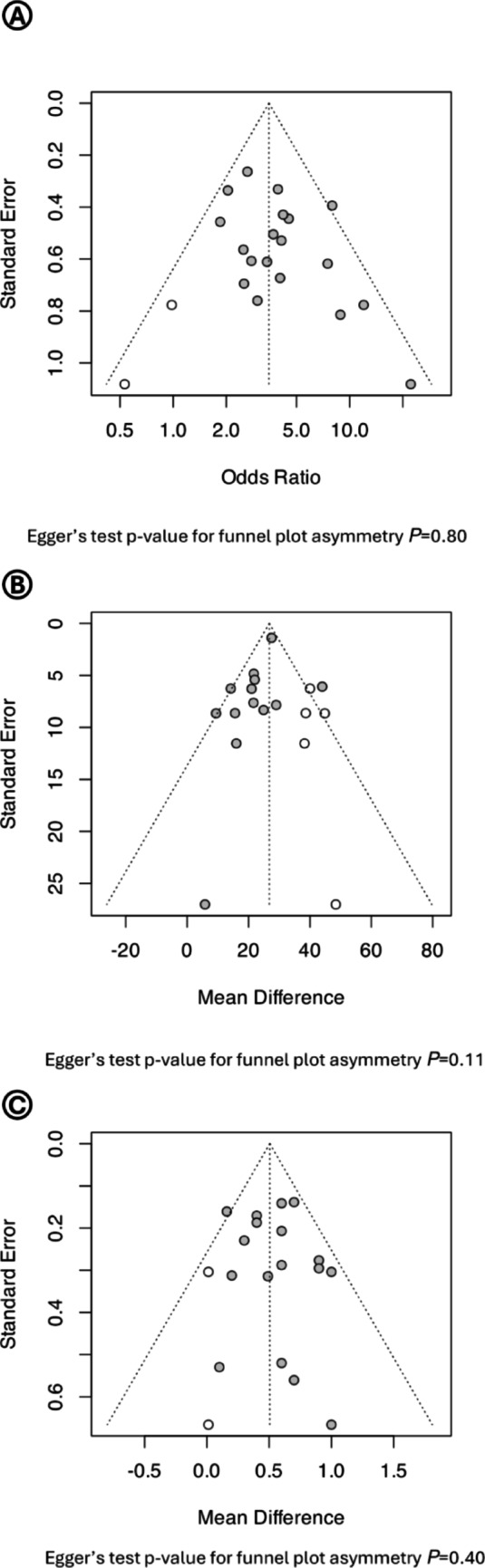
Trim-and-fill funnel plot and Egger’s test for publication bias results for (**A**) diabetes mellitus, (**B**) fasting plasma glucose, and (**C**) glycated hemoglobin

Most studies had a low risk of attrition and reporting bias. However, three studies had a high risk of attrition bias [45, 50, 52], while five had high risk of reporting bias [36, 45–48] (Supplementary Table S1). Common-effect and random-effect models produced similar results, and leave-one-out meta-analysis confirmed the robustness of the findings across all evaluated primary outcomes (i.e., DM, FPG, and HbA_1c_) (Fig. 4). Trim-and-fill funnel plot as well as the Egger’s test suggested limited influence of publication bias on the reported outcomes (Fig. 5).

## Discussion

This large systematic review including data from both clinical trials and real-life scenarios is the first comprehensive synthesis of the effects of PasiLAR on lipid and glucose metabolism in patients with acromegaly. Specifically, patients with acromegaly treated with PasiLAR for at least 6 months had significantly higher levels of HDL-C, whereas no significant changes were found in TC, LDL-C, or TG levels compared to baseline. Moreover, PasiLAR treatment was associated with an increase in FPG, HbA_1c_, and the proportion of patients with DM.

Impairment of lipid and glucose metabolism is a common complication in acromegaly. The GH excess impairs insulin signaling and promotes lipolysis, leading to increased levels of free fatty acids which contributes to insulin resistance and development of DM [11, 22]. Acromegaly is also associated with dyslipidemia, especially hypertriglyceridemia and low levels of HDL-C [7, 11]. There is conflicting data on TC and LDL-C, whose trends seem to be unchanged during the course of the disease in most studies [11, 22]. Conversely, there is more consensus on denser and more atherogenic LDL particles [11, 22], namely Lp(a), lipid molecules composed of an LDL molecule bound to a portion of apolipoprotein(a), which are associated with atherosclerotic cardiovascular disease and an increased risk of serious cardiovascular affections in the general population [68–70]. However, the direct influence of dyslipidemia on atherosclerotic cardiovascular disease in acromegaly is still debated [21, 22].

This meta-analysis shows, for the first time, that HDL-C levels increased by a mean of 6.2 mg/dL after treatment with PasiLAR and this may be due to the improved disease control. Considering that statins are able to increase HDL cholesterol by 5–10% (e.g.,+2–4 mg/dL) [71–73], we can define the increase in HDL-C values induced by Pasireotide LAR as clinically relevant. Higher HDL-C levels have been associated with a lower risk of cardiovascular mortality and non-fatal myocardial infarction in the general population [68], but whether this increase in HDL-C has any impact on cardiovascular risk in patients with acromegaly remains unknown. This meta-analysis examined the effects of PasiLAR of glucose metabolism in the largest cohort of patients with acromegaly to date, including 19 studies and a total of 896 patients. Specifically, after at least 6 months of treatment with PasiLAR, FPG increased by a mean of 23.4 mg/dL, HbA_1c_ by 0.5%, and the risk of DM rose 3.7-fold (*p* < 0.01). Our results align with findings from key studies, including the randomized, phase 3, PAOLA Study [40, 60] as well as extension studies [48, 58, 62, 64, 65], demonstrating that PasiLAR is effective in disease control but with an unfavorable effect on glucose metabolism. Moreover, our results align with two previous meta-analyses. Aliyeva and al. [74] showed hyperglycemia in 29.6% and new-onset DM in 23.4% of 590 patients with acromegaly after 12 months of treatment. Biagetti and colleagues [28]reported significant increases in FPG and HbA_1c_ in 409 patients.

Available suggests that PasiLAR-induced hyperglycemia has a rapid onset after treatment start and FPG reaches a peak within 1–3 months and is reversible after treatment discontinuation [1]. Limited research exists on effective treatments for diabetes in acromegaly patients treated with PasiLAR. Some studies have suggested that PasiLAR-induced hyperglycemia is manageable with common antidiabetic medications such as metformin, sitagliptin, liraglutide, and insulin [47]. Incretin-based drugs such as dipeptidyl peptidase-4 inhibitors and glucagon-like peptide-1 receptor agonists seem to be particularly effective [39, 75].

It has been shown that some baseline factors influence the occurrence of hyperglycemia under PasiLAR treatment such as older age, the presence of impaired glucose tolerance, elevated HbA_1c_, a history of dyslipidemia or hypertension, and body mass index ≥ 30 kg/m^2^ [76]. In addition, recent findings suggest that, after dose reduction of PasiLAR, glucose levels and HbA_1c_ declined in a majority of patients, suggesting a possible improvement in glycemic control without the need for definitive discontinuation of the drug [67, 77, 78].

Regarding combination treatment, only one study (6 patients) by Chiloiro and colleagues [38] included data on glucose metabolism in patients treated with both PasiLAR and PegV. Combination treatment controlled the disease in 83.3% of cases and was associated with better metabolic outcomes and a reduction of HbA_1c_ as compared to PasiLAR alone, suggesting a possible balancing role of PegV on glucose deterioration.

Combination treatment (PasiLAR + PegV) was also explored in the PAPE Study [39], where patients with acromegaly controlled with first-generation SSTR ligands and PegV were switched to PasiLAR with or without PegV. Since it was not possible to extrapolate separated glycemic outcomes for the cohort that received PasiLAR monotherapy from the cohort that received combination of PasiLAR + PegV, this study and its extension [63] were not included in the quantitative analysis. In that trial, hyperglycemia-related adverse events occurred in 88.5% of patients, with DM prevalence rising from 32.8 to 68.9%. These results were likely influenced by older age and pre-diabetic conditions at baseline. Furthermore, reductions in PegV doses during treatment probably exacerbated glucose metabolism abnormalities.

Some limitations of this meta-analysis must be acknowledged. Publication bias cannot be excluded as studies with null or negative results may be under-reported, though efforts were made to minimize this through a comprehensive literature search including gray literature (unpublished studies, abstracts, etc.). Inconsistencies in reporting may also have been caused by different definitions of hyperglycemia and DM between studies. Sample size limitations also prevented us from undertaking the pre-planned subgroup analyses on quality of study and sex. Missing data on key glucose-related metrics such as 2-hour oral glucose tolerance test glucose, blood pressure, fasting plasma insulin, and body mass index limited the analysis to DM status, FPG, and HbA_1c_. Similarly, data on lipid metabolism were found only in two studies, constituting another limitation to this meta-analysis, with only HDL-C showing significant results, while information on LDL-C, TC, and TG remained insufficient for robust conclusions, and data on Lp(a) was missing. These gaps highlight the need for more comprehensive and standardized studies to fully elucidate the metabolic effects of PasiLAR, particularly concerning lipid metabolism in acromegaly.

In conclusion, this systematic review and meta-analysis is the first comprehensive evaluation of the effects of PasiLAR treatment on both lipid and glucose metabolism in patients with acromegaly. PasiLAR treatment in acromegaly was associated with a significantly higher level of HDL-C, and no changes in TG, TC, and LDL-C. Data on lipid metabolism was however scanty, highlighting the need for more comprehensive studies to fully elucidate the impact of PasiLAR on lipid profile in acromegaly. In addition, treatment with PasiLAR was associated with higher FPG, HbA_1c_, and frequency of DM compared to baseline. Our results highlight the importance of a personalized approach to the treatment of acromegaly with a careful examination of metabolic status. Identification of risk factors for hyperglycemia and dyslipidemia, attentive glucose monitoring, and early detection of worsening glycemic control are of crucial importance for reducing the risk of DM in acromegaly.

## Electronic supplementary material

Below is the link to the electronic supplementary material.

Supplementary Material 1

## Data Availability

Original data generated and analyzed during this study are included in this published article or in the data repositories listed in the Reference section.
