# Supplementary material for: Impact of pasireotide on lipid and glucose metabolism in patients with acromegaly: a systematic review and meta-analysis
Source: J Endocrinol Invest. 2025 Jul 7;48(12):2799–812. doi: 10.1007/s40618-025-02642-0 (PMC12640336; doi:10.1007/s40618-025-02642-0)
Supplement: Supplementary file 1 — Supplementary Material 1 [file 40618_2025_2642_MOESM1_ESM.docx]

**Impact of pasireotide on lipid and glucose metabolism in patients with acromegaly: a systematic review and meta-analysis**

Costanza F, Basile C, Chiloiro S, et al

**Supplemental Fig S1** Search strategy

Search: Date: 2024-04-11

Databases: Cochrane Library(Wiley), Embase (Elsevier), PubMed, Web of Science (Clarivate)

Total number of hits: 5438
Cochrane Library (*n* = 198), Embase (*n* = 2706), PubMed (*n* = 1413), Web of Science (*n* = 1121)

Before deduplication: 5438
After deduplication: 3335 (duplicates [*n*=2103])

Updated search: Date: 2024-09-11

Additional number of hits: 189 (limit to years: 2024–2025)
Databases: Cochrane Library (*n* = 4), Embase (*n* = 77), PubMed (*n* = 63), Web of Science (*n* = 45)

Before deduplication: 189
After deduplication: 106 (duplicates [*n* = 83])

Comments:
One retracted article retrieved:
Yu BX, Zhang ZS, Song H, Chi YC, Shi CL, Xu M. RETRACTED (2017) Clinical importance of somatostatin receptor 2 (SSTR2) and somatostatin receptor 5 (SSTR5) expression in thyrotropin-producing pituitary adenoma (TSHoma) (retracted article). *Med Sci Monit* 23:1947–1955

PubMed

| Interface: pubmed.gov  Date of Search: 2024-04-11  Number of hits: 1413  Comment: | Field labels   - [mh] or [mesh] = exploded MeSH term - [tiab] = title, abstract - * = truncation of word for alternate endings NB If truncating a phrase – the truncated term must be the last word in the phrase. |
| --- | --- |
| \| **#** \| Searches \| Results \| \| --- \| --- \| --- \| \| 1 \| (Pasireotide[nm] OR Pasireotide[tw] OR Signifor[tiab] OR SOM 230[tw] OR SOM230[tw] OR somatostatin analog*[tw] OR Somatostatin/analogs and derivatives[mh] OR somatostatin receptor ligand*[tw]) \| 10,018 \| \| 2 \| (Acromegaly[mh] OR acromegaly[tiab] OR Somatotropin Hypersecretion Syndrome*[tiab] OR Inappropriate GH Secretion Syndrome*[tiab] OR Inappropriate Growth Hormone Secretion Syndrome*[tiab] OR GH-secreting pituitary adenoma*[tiab]) \| 11,918 \| \| 3 \| #1 AND #2 \| 1807 \| \| 4 \| Filters: from 2000 – 2024 \| 433 \| \| 5 \| NOT ("Animals"[Mesh] NOT ("Animals"[Mesh] AND "Humans"[Mesh]) \| 5,209,662 \| \| 6 \| #4 NOT #5 \| 1413 \| \|  \|  \|  \| | |

Cochrane Library

| Interface: Cochrane Library  Date of Search: 2024-04-11  Number of hits: 198 | Field labels   - ti,ab,kw = title, abstract and author keywords - NEAR/x = within x words, regardless of order - * = truncation of word for alternate endings |
| --- | --- |
| \| **#** \| **Searches** \| **Results** \| \| --- \| --- \| --- \| \| 1 \| (Pasireotide:ti,ab OR Signifor:ti,ab OR "SOM 230":ti,ab OR SOM230:ti,ab OR ("somatostatin" NEXT analog*):ti,ab OR ("somatostatin receptor" NEXT ligand*):ti,ab) \| 1010 \| \| 2 \| MeSH descriptor: [Somatostatin] explode all trees and with qualifier(s): [analogs & derivatives - AA] \| 285 \| \| 3 \| #1 OR #2 \| 1071 \| \| 4 \| (acromegaly:ti,ab OR ("Somatotropin Hypersecretion" NEXT Syndrome*):ti,ab OR ("Inappropriate GH Secretion" NEXT Syndrome*):ti,ab OR ("Inappropriate Growth Hormone Secretion" NEXT Syndrome*):ti,ab OR ("GH-secreting pituitary" NEXT adenoma*):ti,ab) \| 496 \| \| 5 \| MeSH descriptor: [Acromegaly] explode all trees \| 308 \| \| 6 \| #4 OR #5 \| 532 \| \| 7 \| #3 AND #6 \| 227 \| \| 8 \| #7 with Publication Year from 2000 to 2024, with Cochrane Library publication date from Jan 2000 to Dec 2024, in Trials \| 198 \| \|  \|  \|  \| | |

Web of Science Core Collection

| Interface: Clarivate Analytics  Date of Search: 2024-04-11  Number of hits: 1121 | Field labels   - TS/Topic = title, abstract, author keywords and Keywords Plus - NEAR/x = within x words, regardless of order - * = truncation of word for alternate endings |
| --- | --- |
| \| **#** \| **Searches** \| **Results** \| \| --- \| --- \| --- \| \| 1 \| TS=(Pasireotide OR Signifor OR "SOM 230" OR SOM230 OR "somatostatin analog*" OR "Somatostatin derivative*" OR "somatostatin receptor ligand*") \| 10,816 \| \| 2 \| TS=(Acromegaly OR "Somatotropin Hypersecretion Syndrome*" OR "Inappropriate GH Secretion Syndrome*" OR "Inappropriate Growth Hormone Secretion Syndrome*" OR "GH-secreting pituitary adenoma*") \| 8497 \| \| 3 \| #1 AND #2 \| 1830 \| \| 4 \| #1 AND #2 and Article or Meeting Abstract or Proceeding Paper or Retracted Publication or Early Access or Correction (Document Types) \| 1423 \| \| 5 \| #1 AND #2 and Article or Meeting Abstract or Proceeding Paper or Retracted Publication or Early Access or Correction (Document Types) and 2000 or 2001 or 2002 or 2003 or 2004 or 2005 or 2006 or 2007 or 2008 or 2009 or 2010 or 2012 or 2013 or 2014 or 2015 or 2016 or 2017 or 2018 or 2019 or 2020 or 2021 or 2022 or 2023 or 2024 or 2011 (Publication Years) \| 1121 \| \|  \|  \|  \| | |

Embase

| Interface: Elsevier  Date of Search: 2024-04-11  Number of hits: 2706  Comment: use ' for phrase searching | Field labels   - /exp = exploded Emtree term - /de = non exploded Emtree term - :ti,ab,kw = title, abstract and author keywords - NEAR/n = within x words, regardless of order - * = truncation of word for alternate endings - * = variable wildcard, i.e one or more letters - ' = phrase |
| --- | --- |
| \| **#** \| **Searches** \| **Results** \| \| --- \| --- \| --- \| \| 1 \| (Pasireotide/exp OR Pasireotide:ti,ab,kw OR Signifor:ti,ab,kw OR 'SOM 230':ti,ab,kw OR SOM230:ti,ab,kw OR 'somatostatin analog*':ti,ab,kw OR 'somatostatin derivative'/exp OR 'somatostatin derivative*':ti,ab,kw OR 'somatostatin receptor ligand'/exp OR 'somatostatin receptor ligand*':ti,ab,kw) \| 67,848 \| \| 2 \| (Acromegaly/exp OR acromegaly:ti,ab,kw OR 'Somatotropin Hypersecretion Syndrome*':ti,ab,kw OR 'Inappropriate GH Secretion Syndrome*':ti,ab,kw OR 'Inappropriate Growth Hormone Secretion Syndrome*':ti,ab,kw OR 'GH-secreting pituitary adenoma*':ti,ab,kw) \| 17,065 \| \| 3 \| #1 AND #2 \| 4836 \| \| 4 \| #3 AND ('article'/it OR 'article in press'/it OR 'conference abstract'/it OR 'conference paper'/it OR 'erratum'/it) \| 3456 \| \| 5 \| #4 AND (2000:py OR 2001:py OR 2002:py OR 2003:py OR 2004:py OR 2005:py OR 2006:py OR 2007:py OR 2008:py OR 2009:py OR 2010:py OR 2011:py OR 2012:py OR 2013:py OR 2014:py OR 2015:py OR 2016:py OR 2017:py OR 2018:py OR 2019:py OR 2020:py OR 2021:py OR 2022:py OR 2023:py OR 2024:py) \| 2761 \| \| 6 \| #5 NOT ([animals]/lim NOT [humans]/lim) \| 2706 \| \|  \|  \|  \| | |

**Supplemental Table S1** Bias assessment of selected studies for quantitative analysis. Abbreviation: NE, not evaluable

| First author, year (reference no.) | Selection bias | Performance bias | Detection bias | Attrition bias | Reporting bias | Other bias |
| --- | --- | --- | --- | --- | --- | --- |
| Gadelha et al (2014) (40] | ? | ? | ? | – | - | – |
| Petersenn et al (2014) [56] | NE | NE | NE | – | – | – |
| Sheppard et al (2015) [48] | ? | ? | ? | – | + | – |
| Bronstein et al (2016) [57] | NE | NE | NE | – | – | – |
| Fleseriu et al (2016) [41] | NE | NE | NE | – | - | – |
| Tahara et al (2017) [42] | ? | ? | ? | – | – | – |
| Shimon et al (2018) [43] | NE | NE | NE | – | – | – |
| Lasolle et al (2019) [45] | NE | NE | NE | – | - | – |
| Gadelha et al (2020) [44] | NE | NE | NE | – | - | – |
| Chiloiro et al (2021) [38] | NE | NE | NE | – | + | – |
| Stelmachovska-Banas et al (2021) [51] | NE | NE | NE | – | – | – |
| Witek et al. (2021) [49] | NE | NE | NE | – | + | – |
| Wolf et al (2022) [50] | NE | NE | NE | – | + | – |
| Corica et al (2023) [52] | NE | NE | NE | + | – | – |
| Gadelha et al (2023) [58] | NE | NE | NE | – | – | – |
| Ruiz et al (2023) [37] | NE | NE | NE | – | + | – |
| Araujo-Castro 2024) [59] | NE | NE | NE | – | + | – |
| Favero et al (2024) [53] | NE | NE | NE | – | - | – |
| Pirchio et al (2024) [54] | NE | NE | NE | + | – | – |
| Urbani et al (2024) [55] | NE | NE | NE | – | – | – |

*Note:* Full references are cited in the main manuscript.

– indicates low risk; + indicates high risk; ? indicates an unclear risk of bias.
